# Hypermethylation of the Gene Coding for PGC-1α in Peripheral Blood Leukocytes of Patients With Parkinson’s Disease

**DOI:** 10.3389/fnins.2020.00097

**Published:** 2020-02-26

**Authors:** Xiaodong Yang, Shaoqing Xu, Yiwei Qian, Xiaoqin He, Shengdi Chen, Qin Xiao

**Affiliations:** Department of Neurology, Ruijin Hospital Affiliated with the School of Medicine, Shanghai Jiao Tong University, Shanghai, China

**Keywords:** Parkinson’s disease, DNA methylation, PGC-1α, variants, mRNA expression

## Abstract

Decreased expression of peroxisome proliferator-activated receptor gamma coactivator-1α (PGC-1α) is implicated in the pathophysiology of Parkinson’s disease (PD). However, our understanding of the mechanism regulating the PGC-1α expression is still limited. We sought to determine whether the epigenetic modification of *PPARGC1A* (the gene encoding PGC-1α) could account for its diminished expression. We performed a study of *PPARGC1A* risk-SNP genotypes, methylation level, and the expression in blood from 171 subjects. The mean DNA methylation level of *PPARGC1A* intron 1 in patients with PD was higher than that in the controls (7.18 ± 1.74 vs. 6.36 ± 1.28, *P* = 0.007). A detailed comparison of the DNA methylation level at each CpG site showed that CpG_1, CpG_13.14, CpG_17.18, and CpG_20 were significantly hypermethylated in patients with PD. There was a significant negative correlation between *PPARGC1A* methylation and expression level (*R* = −0.404, *P* < 0.001). We found no correlations between the *PPARGC1A* methylation level and the clinical features, while the CpG_13.14 site methylation level was positively correlated with H&Y stage (*R* = 0.246, *P* = 0.020) and was increased in people carrying the rs2970848 AA genotype compared with that in carriers of the AG/GG genotype (7.27 ± 1.86 vs. 6.65 ± 1.92, *P* = 0.032). Our results support a link between *PPARGC1A* methylation, gene expression, and variability, which indicated that a novel epigenetic regulatory mechanism controlling *PPARGC1A* expression influences PD pathogenesis.

## Introduction

Parkinson’s disease (PD) is a chronic, progressive neurodegenerative disorder. It is estimated that by 2030, Chinese PD patients will increase to 4.94 million, accounting for a half of the PD patients worldwide (Li et al., 2019). Recent advances have helped delineate the pathogenetic mechanisms, yet the etiology and pathogenesis of PD in most individuals remain obscure. Over recent years, extensive genetic screening of families with PD has identified several mutations associated with PD. Additionally, environmental exposures have been suggested to play a crucial role in the etiological process of PD. It has been accepted that in most patients, the disease is caused by complicated interactions between genetic and environmental risk factors (Gao and Hong, 2011). Environmental risk factors such as pesticides, solvents, heavy metals, industrialized nutrition, and other pollutants are considered to affect disease risk through epigenetic mechanisms. This raises the possibility that epigenetic modifications, including DNA methylation, histone tail modifications, and chromatin remodeling, as well as small and long non-coding RNAs, can have a fundamental role in the gene–environment interactions that are related to PD (Miranda-Morales et al., 2017).

Epigenetic modifications are chemical modifications of chromatin or its regulatory proteins that do not change the underlying genomic sequence. These modifications can modulate multiple levels of gene expression, from direct modifications of the DNA and histone tails, regulating the level of transcription, to interactions with mRNAs, regulating the level of translation (Lardenoije et al., 2015). DNA methylation is the most intensively studied epigenetic mechanism involved in the regulation of gene transcription. Dense DNA methylation at promoters is associated with gene repression, whereas unmethylated promoters are mostly associated with gene activation (Jones, 2012; Yoshino et al., 2017). It is proposed that epigenetic mechanisms provide a bridge between the genes and the environment and may help to improve our understanding of the etiology of PD.

There is growing interest in exploring the role of DNA methylation in patients with PD. Methylation patterns were not considered as a new source of biomarkers for PD, but these were also associated with PD motor and cognitive progression (Chuang et al., 2019). The investigation of significant DNA methylation changes and gene expression analyses of PD-associated genes has produced contradictory and inconsistent results. It has been shown that the *SNCA* CpG island is hypomethylated in the brain tissue and in the blood of patients with PD, and there was a close link between *SNCA* methylation levels and *SNCA* expression (Pihlstrøm et al., 2015). Decreased methylation at *SNCA* intron 1 might contribute to the deregulation of α-syn expression (Jowaed et al., 2010). However, some studies found no evidence for DNA methylation changes within the *SNCA* promoter region of patients with PD (Richter et al., 2012; Guhathakurta et al., 2017). Moreover, interindividual genetic variants can frequently be associated with DNA methylation differences at distinct CpG sites. *SNCA* rs3756063 showed a significant correlation with the DNA methylation state of *SNCA* intron 1 in both the brain and blood samples of PD patients (Pihlstrøm et al., 2015).

Peroxisome proliferator-activated receptor gamma coactivator-1α (PGC-1α) is a transcription coactivator for nuclear receptors and has a key integratory role in the transcriptional control of cellular energy metabolism, microglial plasticity, and mitochondrial function. Recent studies have indicated that PGC-1α impairment plays a role in PD pathogenesis (Eschbach et al., 2015; Yang et al., 2017). Reduced expression of PGC-1α and increased *PPARGC1A* promoter methylation in the brains of patients with PD were reported in a subsample of sporadic PD substantia nigra samples compared to those in samples from 10 age-matched controls (Su et al., 2015). However, access to brain samples for research is limited, and the number of samples is decreased. Recently, we found that *PPARGC1A* expression was decreased in the peripheral blood leukocytes (PBLs) of patients with PD and was negatively correlated with disease severity (Yang et al., 2018). This provided evidence that PGC-1α was implicated in the pathophysiology of PD. A study has found concordant DNA methylation patterns between blood and brain tissue DNA in PD. It has been demonstrated that DNA methylation changes in PBLs correlate with those of the brain methylome and peripheral blood, which is a more easily accessible tissue and is a surrogate for brain tissue (Masliah et al., 2013). Thus, in this study, we sought to determine the methylation level of *PPARGC1A* in peripheral blood and whether the epigenetic modification of *PPARGC1A* could account for its diminished expression in the peripheral blood of patients with PD.

In our study, we performed an analysis of *PPARGC1A* risk-SNP genotypes, methylation levels, and mRNA expression in peripheral blood from patients with PD and from healthy controls. We aimed to explore the hypothesis that the *PPARGC1A* variants are associated with its promoter methylation level, thus affecting its expression.

## Materials and Methods

### Clinical Subjects

A total of 90 Chinese PD patients and 81 age- and gender-matched controls of Han ancestry were included in the study. The PD patients were recruited from the outpatient clinic at the Department of Neurology, Ruijin Hospital, affiliated with Shanghai Jiao Tong University. PD diagnosis was carried out, using the United Kingdom PD Society Brain Bank criteria (Hughes et al., 1992), by at least two movement disorder specialists. The control group exhibiting no disease symptoms was recruited from the Health Examine Center. Participants who have major organ dysfunction, neurological disease, or diabetes were excluded from the study. For each participant, demographic and clinical data were recorded, including demographic information, Hoehn and Yahar (H&Y) stage, Unified Parkinson’s Disease Rating Scale (UPDRS) scores, Non-motor Symptoms Questionnaire for Parkinson’s disease, Hamilton Anxiety Scale (HAMA), Hamilton Depression Scale (HAMD), and Montreal Cognitive Assessment (MoCA). Levodopa equivalent doses (LED) were calculated by using a method reported in a previous study (Tomlinson et al., 2010). All of the participants signed the informed consent, and the study was approved by the Research Ethics Committee, Ruijin Hospital, affiliated to Shanghai Jiao Tong University School of Medicine.

### DNA/RNA Extraction

Venous whole blood samples (4–5 ml) were collected from PD patients and controls. Genomic DNA was extracted from PBLs by using the conventional phenol/chloroform extraction method. Total RNA was isolated by the standard Trizol method by using QIAamp RNA Blood Mini kit according to the manufacturer’s instructions (QIAGEN, Germantown, MD, United States). DNA and RNA samples were stored in −80°C until use in the experiments.

### DNA Methylation Analyses

**FIGURE 1:**
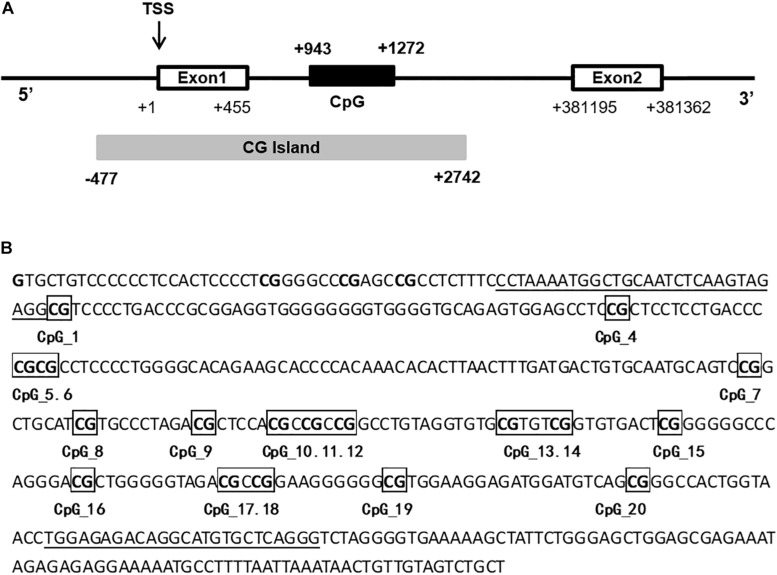
Schematic drawing of the 5’ region of *PPARGC1A*. **(A)** The translational start site is marked in exon 1 by TSS. The white boxes represent exon 1 and exon 2, and the CpG-rich region is depicted as a CpG island (CG Island). The black box represents the CpG island in the intron 1 of *PPARGC1A*. **(B)** Sequence of the amplified fragment of *PPARGC1A* intron 1 and 20 CpG sites (marked with numbers) in this region. Primers are underlined.

Primers for *PPARGC1A* were designed by the EpiDesigner website to cover the regions with the most CpG sites. A 330-bp *PPARGC1A* intron 1 fragment (+ 943 to + 1,272) was analyzed (Figure 1). Due to either low or high mass of the DNA cleavage lengths, 20 of 22 sites in the amplicons generated measurable DNA methylation data. Genomic DNA was bisulfite-converted with the reaction condition as follows: 20 cycles at 95°C for 30 s and 50°C for 15 min. As controls, EpiTect^®^ PCR control DNA (Qiagen, Hilden, Germany) was used in the experiments. The conversion efficiency was >99%. The sequence of *PPARGC1A* was queried from the University of California—Santa Cruz genome biological information network^1^. Bisulfite-specific primers were used to apply bisulfite-treated DNA and generate PCR amplicons. The PCR amplicons were then spotted on 384-element silicon matrix-preloaded chips (SpectroCHIP; Sequenom, San Diego, CA, United States). Mass spectra were collected by using the Mass-ARRAY compact matrix-assisted laser desorption/ionization time-of-flight (MALDI-TOF) system (Sequenom), and the methylation ratios of the spectra were generated by using the EpiTYPER software v1.0 (Sequenom). The level of DNA methylation was determined as the percentage of methylated CpG sites. For each subject, the methylation level (methylated CpG/total CpG) at the individual CpG site and the mean methylation level at all 20 CpG sites were calculated.

### Genotype Analyses

The detection of SNPs was performed by using the MassARRAY system (Sequenom, San Diego, CA, United States) using chip-based MALDI-TOF mass spectrometry technology. Primers for PCR and single-base extensions were designed by using the Sequenom Mass-ARRAY Assay Design 3.0 Software (Sequenom, San Diego, CA, United States). The corresponding primers used for each SNP in the present study are listed in Supplementary Table S1.

### cDNA Synthesis and Quantitative Real-Time PCR

PBL PPARGC1A expression level was investigated in patients with PD and in healthy controls by using qPCR. Reverse transcription reactions were performed by using a TaKaRa 1st-strand kit (Dalian, China) according to the manufacturer’s protocol. The synthesized cDNA was subjected to real-time PCR assays with specific primers and SYBR Premix Ex Taq qPCR SuperMix (TakaRa, Dalian, China). The sequences of the *PPARGC1A* primers are as follows:

Forward: 5′-TAAACGACTCCGAGAACA-3′
Reverse: 5′-GACCCAAACATCATACCC-3′

The RNA quantities of target genes were calculated by using the Ct method. The final results were normalized and expressed as the fold change compared to the target gene and/or actin.

### Statistical Analyses

All statistical analyses were performed by using the Statistical Package for the Social Sciences (SPSS, version 18.0, IBM Corp., Chicago, IL, United States) for Windows. Continuous data were presented as mean ± standard deviation (SD). Chi-square test or Fisher’s exact test was used to analyze the categorical data between the control group and the PD group. The data were analyzed by *t*-test or one-way analysis of variance (ANOVA) if the data were normally distributed. Non-parametric Mann–Whitney U or Kruskal–Wallis tests were used for continuous variables if the data were not normally distributed. The Pearson’s correlation coefficient was used for correlation analysis. For all analyses, *P* value < 0.05 was defined as a statistically significant difference.

## Results

### *PPARGC1A* Methylation and Expression Levels in Patients With PD and in Controls

**FIGURE 2:**
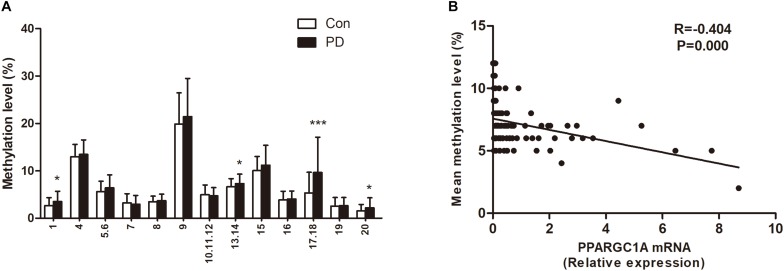
**(A)** Site specific methylation levels of *PPARGC1A* intron 1. *P < 0.05, ***P < 0.001. **(B)** Correlation between the mean DNA methylation level of *PPARGC1A* and the *PPARGC1A* mRNA level. Each plot represents the methylation level of individual.

The demographic and clinical data for all subjects are summarized in Table 1. There was no significant difference in sex or age between patients with PD and healthy controls. The mean DNA methylation level of PPARGC1A intron 1 in patients with PD was higher than that of controls (7.18 ± 1.74 vs. 6.36 ± 1.28, *P* = 0.007). When the effects of age and gender were examined as covariates, there was still a significant difference between the PD patients and the healthy controls (*P* = 0.008). A detailed comparison of DNA methylation level at each CpG site was also performed for the two groups. The results showed that CpG_1, CpG_13.14, CpG_17.18, and CpG_20 were significantly hypermethylated in patients with PD (Figure 2A). In agreement with the results from our previous study, we found that the *PPARGC1A* mRNA level was significantly decreased in patients with PD (2.85 ± 3.30 vs. 0.99 ± 1.61, *P* < 0.001).

**TABLE 1:** Clinical characteristics and levels of *PPARGC1A* methylation and mRNA of Parkinson’s disease patients and controls.

	PD	Control	*P* value
Number	90	81	
Gender (male/female)	43/47	39/42	1.000^a^
Age (years)	64.83 ± 9.62	66.59 ± 9.46	0.231^b^
Age of onset (years)	59.30 ± 11.51		
Disease duration (years)	4.95 ± 4.08		
H&Y stage	2.10 ± 0.99		
UPDRS III	26.39 ± 17.23		
NMS	7.07 ± 4.28		
HAMD	6.91 ± 7.89		
HAMA	7.77 ± 7.77		
MoCA	22.62 ± 5.01		
LED	408.70 ± 246.11		
Mean methylation level (%)	7.18 ± 1.74	6.36 ± 1.28	0.007^c^
*PPARGC1A* expression level	0.99 ± 1.61	2.85 ± 3.30	0.000^c^

*PD, Parkinson’s disease; H&Y stage, Hoehn and Yahr stage; UPDRS, Unified Parkinson’s Disease Rating Scale; NMS, Non-motor Symptoms Questionnaire; HAMD, Hamilton Rating Scale for Depression; HAMA, Hamilton Anxiety Scale; MoCA, Montreal Cognitive Assessment; LED, levodopa equivalent doses.^*a*^*P*-value for chi-square/Fischer’s exact test,^*b*^*P*-value for unpaired *t*-test, ^*c*^*P*-value for Mann–Whitney *U* test.*

### Effect of DNA Methylation on *PPARGC1A* Expression

There was a significant negative correlation between the mean DNA methylation level of *PPARGC1A* and its expression (Figure 2B). The correlation of DNA methylation level at individual CpG sites with *PPARGC1A* mRNA level was also analyzed (Table 2). The results showed that there was a negative correlation between the *PPARGC1A* mRNA level and the CpG_1 (*R* = −0.328, *P* < 0.001), CpG_13.14 (*R* = −0.124, *P* = 0.026), and CpG_17.18 (*R* = −0.327, *P* < 0.001) sites. After correcting for multiple comparisons, there was still a negative correlation between the *PPARGC1A* mRNA level and the mean DNA methylation level in the CpG_1 and CpG_17.18 sites of *PPARGC1A*.

**TABLE 2:** Correlations between *PPARGC1A* methylation level with Parkinson’s disease clinical data.

Clinical parameters	Methylation level			CpG_1			CpG_13.14			CpG_17.18			CpG_20		
					
	*R*	*P*	FDR	*R*	*P*	FDR	*R*	*P*	FDR	*R*	*P*	FDR	*R*	*P*	FDR
Age (years)	0.016	0.834	1.000	0.023	0.769	1.000	0.017	0.824	1.000	−0.103	0.179	1.000	−0.160	**0.037**	0.407
Age of onset (years)	0.076	0.477	1.000	0.073	0.491	1.000	−0.010	0.924	1.000	0.130	0.222	1.000	0.049	0.646	1.000
Disease duration (years)	0.006	0.957	1.000	−0.144	0.176	1.000	0.014	0.899	1.000	−0.025	0.815	1.000	−0.145	0.173	1.000
H&Y stage	0.167	0.116	1.000	0.044	0.683	1.000	0.246	**0.020**	0.220	0.027	0.801	1.000	−0.120	0.261	1.000
UPDRS III	0.123	0.248	1.000	−0.032	0.762	1.000	0.158	0.136	1.000	0.073	0.494	1.000	−0.194	0.067	0.737
NMS	−0.109	0.309	1.000	−0.006	0.955	1.000	0.043	0.685	1.000	−0.158	0.138	1.000	−0.013	0.906	1.000
HAMD	−0.053	0.622	1.000	−0.055	0.606	1.000	0.051	0.631	1.000	−0.107	0.315	1.000	0.052	0.626	1.000
HAMA	−0.061	0.566	1.000	−0.102	0.337	1.000	0.077	0.473	1.000	−0.156	0.141	1.000	0.026	0.807	1.000
MoCA	−0.095	0.375	1.000	0.023	0.831	1.000	−0.145	0.173	1.000	−0.069	0.520	1.000	−0.002	0.983	1.000
LED	0.023	0.827	1.000	0.086	0.422	1.000	0.010	0.922	1.000	−0.113	0.288	1.000	−0.047	0.658	1.000
*PPARGC1A* expression level	−0.404	**0.000**	**0.000**	−0.328	**0.000**	**0.000**	−0.124	**0.026**	0.286	−0.327	**0.000**	**0.000**	−0.145	0.059	0.649

*Significant results are highlighted in bold. PD, Parkinson’s disease; H&Y stage, Hoehn and Yahr stage; UPDRS, Unified Parkinson’s Disease Rating Scale; NMS, Non-motor Symptoms Questionnaire; HAMD, Hamilton Rating Scale for Depression; HAMA, Hamilton Anxiety Scale; MoCA, Montreal Cognitive Assessment; LED, levodopa equivalent doses. FDR, false discovery rate. *P*-value for Pearson’s correlation analysis.*

### Correlations Between Methylation Level and PD Clinical Data

We then evaluated the correlations between methylation level and clinical features, including age, age of disease onset, disease duration, H&Y stage, UPDRS III scores, HAMA, HAMD, MoCA scores, and LED. None of these factors was associated with the mean methylation level. However, the CpG_13.14 site methylation level had a positive correlation with H&Y stage (*R* = 0.246, *P* = 0.020). We also found a weak negative correlation between the CpG_20 site methylation level and age (*R* = −0.160, *P* = 0.037). However, after correcting for multiple comparisons, these correlations disappeared (Table 2).

### Effect of *PPARGC1A* Variants on DNA Methylation

There was no significant difference in the genotype distribution of *PPARGC1A* between patients with PD and controls (Supplementary Table S2). To determine the potential effect of *PPARGC1A* polymorphism on the DNA methylation of *PPARGC1A* intron 1, we examined the association of *PPARGC1A* methylation level with different *PPARGC1A* genotypes. The *PPARGC1A* mean methylation level did not differ between different *PPARGC1A* genotypes (Table 3). The *PPARGC1A* CpG_13.14 site DNA methylation level was increased in people carrying the rs2970848 AA genotype compared with that in carriers of the AG/GG genotype (7.27 ± 1.86 vs. 6.65 ± 1.92, *P* = 0.032). A subgroup analysis showed that this result was mainly due to the patients with PD (7.73 ± 1.84 vs. 6.71 ± 2.10, *P* = 0.016). The *PPARGC1A* rs2970870 AA + GG genotype showed significantly lower methylation levels than the AG genotype at CpG-1 (2.71 ± 1.84 vs. 3.38 ± 2.10, *P* = 0.034). This phenomenon was also found in the control subjects (2.22 ± 1.48 vs. 3.00 ± 1.80, *P* = 0.040) but not in the patients with PD. The *PPARGC1A* CpG_20 site DNA methylation level was increased in people carrying the rs6821591 CT + CC genotype compared with that in carriers of the TT genotype (2.15 ± 2.05 vs. 1.55 ± 1.45, *P* = 0.033), however, there was no positive result in the subgroup analysis.

**TABLE 3:** *PPARGC1A* methylation level among different SNPs genotypes.

SNPs genotypes		rs2970848			rs2970870			rs6821591		
				
		AA	AG + GG	*P*	AG	AA + GG	*P*	TT	CT + CC	*P*
Mean *PPARGC1A* methylation level (%)	PD	7.29 ± 1.59	7.02 ± 1.94	0.473	7.30 ± 1.76	6.98 ± 1.72	0.398	7.27 ± 1.64	7.12 ± 1.80	0.688
	Con	6.50 ± 1.30	6.23 ± 1.27	0.342	6.43 ± 1.36	6.28 ± 1.20	0.610	6.52 ± 1.39	6.18 ± 1.15	0.236
	Total	6.94 ± 1.52	6.61 ± 1.66	0.167	6.91 ± 1.64	6.62 ± 1.50	0.239	6.85 ± 1.54	6.74 ± 1.63	0.641
CpG_1	PD	3.71 ± 2.26	3.24 ± 2.10	0.313	3.68 ± 2.28	3.24 ± 2.05	0.356	3.52 ± 2.29	3.51 ± 2.16	0.989
	Con	2.65 ± 1.78	2.66 ± 1.64	0.982	3.00 ± 1.80	2.22 ± 1.48	**0.040**	2.43 ± 1.82	2.90 ± 1.54	0.216
	Total	3.25 ± 2.12	2.94 ± 1.88	0.312	3.38 ± 2.10	2.71 ± 1.84	**0.034**	2.91 ± 2.10	3.26 ± 1.94	0.256
CpG_13.14	PD	7.73 ± 1.84	6.71 ± 2.10	**0.016**	7.27 ± 2.06	7.35 ± 1.95	0.847	7.24 ± 1.77	7.33 ± 2.15	0.837
	Con	6.67 ± 1.73	6.59 ± 1.76	0.818	6.84 ± 1.76	6.36 ± 1.69	0.215	6.95 ± 1.74	6.28 ± 1.69	0.082
	Total	7.27 ± 1.86	6.65 ± 1.92	**0.032**	7.08 ± 1.93	6.84 ± 1.88	0.427	7.08 ± 1.75	6.91 ± 2.03	0.556
CpG_17.18	PD	9.44 ± 6.82	9.95 ± 8.31	0.752	10.46 ± 8.53	8.32 ± 5.03	0.187	10.12 ± 7.68	9.39 ± 7.36	0.654
	Con	4.60 ± 4.28	6.07 ± 4.33	0.128	5.78 ± 5.06	4.81 ± 3.23	0.320	5.40 ± 4.24	5.28 ± 4.51	0.900
	Total	7.34 ± 6.30	7.94 ± 6.80	0.550	8.38 ± 7.53	6.51 ± 4.53	0.066	7.48 ± 6.40	7.72 ± 6.64	0.813
CpG_20	PD	2.00 ± 1.62	2.47 ± 2.66	0.298	2.43 ± 2.47	1.82 ± 1.31	0.191	1.82 ± 1.47	2.42 ± 2.41	0.195
	Con	1.50 ± 1.41	1.56 ± 1.34	0.843	1.64 ± 1.50	1.40 ± 1.20	0.408	1.33 ± 1.41	1.74 ± 1.31	0.180
	Total	1.78 ± 1.55	2.00 ± 2.12	0.440	2.08 ± 2.12	1.60 ± 1.27	0.092	1.55 ± 1.45	2.15 ± 2.05	**0.033**

*Significant results are highlighted in bold. *P*-value for Mann–Whitney *U* test.*

## Discussion

Parkinson’s disease is the second most common chronic neurodegenerative disease in the elderly population. The causes of most cases of neurodegenerative diseases remain largely unknown. The epigenome is responsible for the molding and the three-dimensional structure of the genomic material in the cell nucleus. It provides a bridge between the genes and the environment, and it may help to improve our understanding of the etiology of PD (Jakubowski and Labrie, 2017). Recent evidence revealed a reduced expression of PGC-1α and downstream-regulated nuclear-encoded respiratory complex genes in the affected brain tissue and PBLs from patients with PD (Zheng et al., 2010; Eschbach et al., 2015; Yang et al., 2018). The concept of epigenetic regulation of gene expression is over 70 years old; DNA methylation is the most studied epigenetic modification and is known to alter gene expression in a heritable manner (Sweatt, 2013). In our study, we selected and focused on the gene coding for PGC-1α, and we investigated whether the downregulation of *PPARGC1A* is influenced by epigenetic factors. We further investigated the relationship between three common SNPs and the levels of DNA methylation in patients with PD. We found hypermethylation of *PPARGC1A* in the PBLs of patients with PD and a possible regulatory relationship between DNA methylation and its mRNA expression. Moreover, we found that some variants in *PPARGC1A* were associated with its methylation level. Our results elucidate a novel pathway to regulate *PPARGC1A* expression and link the epigenetic mechanisms to a potential PGC-1α role in PD with a focus on potential opportunities for therapeutic intervention.

A number of human studies have described the relationship between *PPARGC1A* DNA methylation and metabolic indicators such as glucose, insulin, and adiposity parameters (Gill and La Merrill, 2017). To date, the epigenetic mechanism in modulating PGC-1α expression has not been fully explored in PD. In the present work, we found that *PPARGC1A* intron 1 was hypermethylated in the PBLs of patients with PD compared with that in controls. This result is consistent with a previous study, which showed that PD was associated with increased methylation of the *PPARGC1A* promoter and reduced expression of PGC-1α in human brain samples (Su et al., 2015). The sequencing results revealed that the majority of the methylated cytosines were not CpG dinucleotides; rather, they were non-canonical cytosine methylated residues. In mammals, the function of non-CpG methylation is currently unknown (Guo et al., 2014). The main reason for this discrepancy is that different CpG sites were analyzed in these two studies. In our study, the CpG island was located in the first intron upstream of the translation initiation site, including 22 CpG sites, while in Su’s study, the sequenced region spanned −358 to −60 relative to the transcription start site of the *PPARGC1A* promoter, and only five CpG sites were analyzed. A sequence analysis of the *SNCA* gene led to the identification of two CpG islands: CpG-1 which starts at the 5′ end of SNCA exon 1 and continues through the exon, and CpG-2 which is located in intron 1. Previous studies found that *SNCA* methylation was mostly found at CpG-2, while *SNCA* CpG-1 was sparsely methylated in blood leukocyte DNA (Tan et al., 2014; Guhathakurta et al., 2017). In addition, previous studies have already suggested that CpG methylation varies depending on age, sex, race, and tissue (de Boni et al., 2011). There is emerging evidence that cytosine methylation, which exists outside of the sequence context of CpG sites (non-CpG methylation: CpA, CpT, and CpC), appears to be most common in embryonic stem cells (Lister et al., 2009) and in adult brain tissue (Guo et al., 2014). In our study, we chose to use PBLs, while in Su’s study, brain samples were used. Tissue-specific regulation of *PPARGC1A* transcription may explain the discrepancy, and additional studies with larger samples in different ethnic populations are necessary to explore *PPARGC1A* DNA methylation in PBLs.

In our study, we examined the methylation and the mRNA levels of *PPARGC1A* at the same time and found a significant negative correlation between the mean DNA methylation level of *PPARGC1A* and the *PPARGC1A* mRNA level, suggesting that *PPARGC1A* DNA methylation, especially at the CpG_1 and CpG_17.18 sites, which were also found to be significantly hypermethylated in patients with PD, may have an effect on *PPARGC1A* transcription. Furthermore, the correlation between *PPARGC1A* DNA methylation and *PPARGC1A* mRNA level was intact after correcting for multiple comparisons. PGC-1α is a critical regulator of mitochondrial biogenesis and respiration, adaptive thermogenesis, glucogenesis, and other metabolic processes, and it is tightly regulated and influenced by both environmental and cell-specific signals (Lindholm et al., 2012). Recent work highlighted a decrease in the expression of PGC-1α and downstream-regulated genes in the SNP of patients with PD, even in the earliest stages of PD. In agreement with the results from our previous study, we found that the *PPARGC1A* mRNA level was significantly decreased in patients with PD compared to those of control samples. In the process of epigenetic modification, hypermethylation is generally associated with decreased expression. A study has found that the hypomethylation of *SNCA* intron 1 could be associated with an increase in *SNCA* expression by promoting the transcription of the gene (Pihlstrøm et al., 2015). Regarding PGC-1α, a study found that free fatty acid-induced hypermethylation of PGC-1α may underlie PGC-1α downregulation (Su et al., 2015). From our results, we confirmed that abnormal *PPARGC1A* methylation might play an important role in regulating PGC-1α expression and in the pathogenesis of PD. Two principal mechanisms, which are not mutually exclusive, are thought to explain the repressive effect of DNA methylation on gene repression. DNA methylation directly interferes with the recognition of transcription factor binding sites or recruits proteins that bind to methylated DNA and thereby mediate silencing. However, other mechanisms may also contribute to PGC-1α downregulation. Mutations in PD genes can affect the PGC-1α levels, PINK1 mutations can result in the impairment of Parkin recruitment, and deficiency of Parkin can lead to the downregulation of PGC-1α through transcriptional repression mediated by PARIS (Shin et al., 2011). α-Synuclein can bind to the *PPARGC1A* promoter and lead to its repression (Eschbach et al., 2015). Apart from gene expression, the activity of PGC-1α is subject to extensive posttranscriptional modifications (Lindholm et al., 2012). Future work is required to delineate the mechanism(s) and magnitude of their respective contribution(s) to *PPARGC1A* DNA methylation and/or the mechanism(s) of regulating PGC-1α expression and its activity.

We found no correlations between methylation level and clinical features. However, the CpG_13.14 site methylation level was positively correlated with H&Y stage. Robust studies with larger sample sizes are required to determine the relationship between different CpG site DNA methylation levels and PD clinical features. Age has been associated with alterations in DNA methylation (Hernandez et al., 2011), and we found a weak negative correlation between CpG_20 site methylation levels and age.

Genetic factors may be involved in the alterations of DNA methylation. Studies have investigated the relationship between genetic variation and CpG methylation in the human brain. The results showed that DNA methylation was frequently heritable, and thousands of SNPs were associated with the methylation of specific CpG sites (Lienert et al., 2011; Wei et al., 2016). In the case of the *SNCA* gene, results have confirmed the association between the *SNCA* rs3756063 variant and the DNA methylation state of *SNCA* intron 1 in both the brain and the blood samples of PD patients (Pihlstrøm et al., 2015). In our study, we evaluated the effects of *PPARGC1A* polymorphism on the methylation levels of *PPARGC1A* intron 1. We did not observe an association between the *PPARGC1A* mean methylation level and the different *PPARGC1A* genotypes. However, the *PPARGC1A* variants may have an effect on CpG site methylation. In particular, the CpG_13.14 site methylation level was increased in people carrying the rs2970848 AA genotype compared with that in carriers of the AG/GG genotype. The CpG_13.14 site methylation level was increased in patients with PD and was positively correlated with H&Y stage. We note that there are several limitations to this study. First, a small number of participants (92 patients with PD and 80 healthy subjects) were recruited. Therefore, it is necessary to conduct a large population study in the future to expand the analysis of DNA methylation in PD-associated genes. Second, gene expression and its regulation vary in different tissues. Additional investigations are required to elucidate the precise relationships between SNPs, DNA methylation, and mRNA expression in different tissues.

Taken together, our results revealed the hypermethylation of *PPARGC1A* in the peripheral blood of patients with PD and a possible regulatory relationship between DNA methylation and its mRNA expression. Epigenetic modifications might be the link between the environmental and genetic risk factors by inducing alterations in gene expression. Our study may help to better understand the mechanisms underlying the associations between SNPs, methylation, and PD.

## Data Availability Statement

All the datasets used and analyzed during the current study are available from the corresponding author on reasonable request.

## Ethics Statement

The studies involving human participants were reviewed and approved by Research Ethics Committee, Ruijin Hospital, affiliated to Shanghai Jiao Tong University School of Medicine. The patients/participants provided their written informed consent to participate in this study.

## Author Contributions

XY and QX designed the study, provided financial support, and revised the manuscript. SC revised the manuscript. XY, SX, YQ, and XH collected the data. XY and SX carried out the genetic analyses. XY and YQ performed the data analysis. XY wrote the manuscript. All of the co-authors contributed to revising the manuscript for intellectual content and approved the final version for publication.

## Conflict of Interest

The authors declare that the research was conducted in the absence of any commercial or financial relationships that could be construed as a potential conflict of interest.

## Footnotes

1 http://genome.ucsc.edu/
