# Supplementary material for: Hypermethylation of the Gene Coding for PGC-1α in Peripheral Blood Leukocytes of Patients With Parkinson’s Disease
Source: Front Neurosci. 2020 Feb 26;14:97. doi: 10.3389/fnins.2020.00097 (PMC7054441; doi:10.3389/fnins.2020.00097)
Supplement: Supplementary file 1 [file Table_1.pdf]

Supplementary Material

Supplementary Table 1

Summary of the primers used for the analysis of the gene polymorphism.

Gene	SNP	First PCR prime (5'-3')	Second PCR prime (5'-3')	UEP SEQ (5'-3')
PPARGC1A	rs2970848	ACGTTGGATGAACCCTCCCTTATGTTGTGC	ACGTTGGATGCTGCAACAGAAACCTAAGTG	GGTCGGCTGGCACTG
	rs2970870	ACGTTGGATGAAAACAATCCCCCAGTTACC	ACGTTGGATGGGAGATGGACAATGAAGAAC	TCCCCCAGTTACCTAAATATA
	rs6821591	ACGTTGGATGCGAACATTTTGAAGTTCTAGG	ACGTTGGATGCAGAATGCGCAAGGGCAAAC	ATTTTGAAGTTCTAGGTTTTAAG
