# Supplementary material for: Hypermethylation of the Gene Coding for PGC-1α in Peripheral Blood Leukocytes of Patients With Parkinson’s Disease
Source: Front Neurosci. 2020 Feb 26;14:97. doi: 10.3389/fnins.2020.00097 (PMC7054441; doi:10.3389/fnins.2020.00097)
Supplement: Supplementary file 2 [file Table_2.docx]

Supplement Table 2
Genotype distribution of *PPARGC1A* in PD patients and healthy controls.

| SNP | Genotype | Frequency in PD patients  (n=90), (%) | Frequency in healthy controls  (n=81), (%) | P value |
| --- | --- | --- | --- | --- |
| rs2970848 | AA  AG+GG | 52(57.8)  38(32.2) | 40(49.4)  41(50.6) | 0.286 |
| rs2970870 | AG  AA+GG | 56(62.2)  34(37.8) | 45(55.6)  36(44.4) | 0.437 |
| rs6821591 | TT  CT+CC | 33(36.7)  57(63.3) | 39(48.1)  42(51.9) | 1.000 |

SNP: Single nucleotide polymorphism. P-value for Chi-square/Fischer’s exact test.
